# Classification of Changes in the Fecal Microbiota Associated with Colonic Adenomatous Polyps Using a Long-Read Sequencing Platform

**DOI:** 10.3390/genes11111374

**Published:** 2020-11-20

**Authors:** Po-Li Wei, Ching-Sheng Hung, Yi-Wei Kao, Ying-Chin Lin, Cheng-Yang Lee, Tzu-Hao Chang, Ben-Chang Shia, Jung-Chun Lin

**Affiliations:** 1 Division of Colorectal Surgery, Department of Surgery, Taipei Medical University Hospital, Taipei Medical University, Taipei 110, Taiwan; poliwei@tmu.edu.tw; 2 Cancer Research Center, Taipei Medical University Hospital, Taipei Medical University, Taipei 110, Taiwan; 3 Translational Laboratory, Department of Medical Research, Taipei Medical University Hospital, Taipei Medical University, Taipei 110, Taiwan; 4 Department of Surgery, College of Medicine, Taipei Medical University, Taipei 110, Taiwan; 5 Graduate Institute of Cancer Biology and Drug Discovery, Taipei Medical University, Taipei 110, Taiwan; 6 College of Medical Science and Technology, Taipei Medical University, Taipei 110, Taiwan; oryx@w.tmu.edu.tw; 7 Department of Laboratory Medicine, Wan Fang Hospital, Taipei Medical University, Taipei 116, Taiwan; 8 Graduate Institute of Business Administration, College of Management, Fu Jen Catholic University, New Taipei City 242062, Taiwan; kyw498762030@gmail.com; 9 Department of Family Medicine, School of Medicine, College of Medicine, Taipei Medical University, Taipei 110, Taiwan; greening1990@gmail.com; 10 Department of Family Medicine, Wan Fang Hospital, Taipei Medical University, Taipei 116, Taiwan; 11 Office of Information Technology, Taipei Medical University, Taipei 106, Taiwan; nathanlee@tmu.edu.tw; 12 Graduate Institute of Biomedical Informatics, College of Medical Science and Technology, Taipei Medical University, Taipei 106, Taiwan; kevinchang@tmu.edu.tw; 13 School of Medical Laboratory Science and Biotechnology, College of Medical Science and Technology, Taipei Medical University, Taipei 110, Taiwan; 14 Pulmonary Research Center, Wan Fang Hospital, Taipei Medical University, Taipei 106, Taiwan

**Keywords:** adenomatous polyp, colorectal cancer, gut microbiota, Oxford nanopore technology

## Abstract

The microbiota is the community of microorganisms that colonizes the oral cavity, respiratory tract, and gut of multicellular organisms. The microbiota exerts manifold physiological and pathological impacts on the organism it inhabits. A growing body of attention is being paid to host–microbiota interplay, which is highly relevant to the development of carcinogenesis. Adenomatous polyps are considered a common hallmark of colorectal cancer, the second leading cause of carcinogenesis-mediated death worldwide. In this study, we examined the relevance between targeted operational taxonomic units and colonic polyps using short- and long-read sequencing platforms. The gut microbiota was assessed in 132 clinical subjects, including 53 healthy participants, 36 patients with occult blood in the gut, and 43 cases with adenomatous polyps. An elevation in the relative abundance of *Klebsiella pneumonia*, *Fusobacterium varium*, and *Fusobacterium mortiferum* was identified in patients with adenomatous polyps compared with the other groups using long-read sequencing workflow. In contrast, the relatively high abundances of *Blautia luti*, *Bacteroides plebeius*, and *Prevotella copri* were characterized in the healthy groups. The diversities in gut microbiota communities were similar in all recruited samples. These results indicated that alterations in gut microbiota were characteristic of participants with adenomatous polyps, which might be relevant to the further development of CRC. These findings provide a potential contribution to the early prediction and interception of CRC occurrence.

## 1. Introduction

A growing body of studies illustrates the putative correlations between the gut microbiota and human health or disease. It is well known that gut dysbiosis is closely related to diarrhea [1], Crohn’s disease [2], and irritable bowel disease [3]. With its high mortality and increasing incidence, colorectal cancer (CRC) is classified as the second leading cause of cancer-associated deaths worldwide [4]. Increasing numbers of studies have documented that lifestyle and dietary intake are critical factors that can change the gut microbiota, which are related to the occurrence and development of CRC [5]. The presence of adenomatous polyps and polyp-induced occult blood (OB) in the gut is widely considered a significant hallmark of colorectal carcinogenesis and was developed as a potential indicator for the early prediction of CRC occurrence [6,7]. Classification of the gut microbial community is not widely applied in clinical settings because of a lack of access to high-throughput sequencing workflow and the need to interpret sequencing results. In addition, expensive and time-consuming high-throughput sequencing restricts the flexibility of clinical applications of the gut microbiota.

Amplicon or short-read sequencing of nine highly variable regions within the bacterial 16S ribosomal (r)RNA gene is most frequently assessed to classify the composition of a microbial profile [8]. As for the gut microbiota, the v3–v4 region is commonly used for accurate taxonomic differentiation [9]. Variable sequencing protocols and analytic workflows are therefore well-developed for 16S rRNA sequencing. Amplicon-based 16S rRNA is sufficient for taxonomic classification of differences to the generic or phylum level but has less power for species-level classification [10]. Oxford Nanopore Technologies (ONT) has developed a non-polymerase chain reaction (PCR)-based sequencing method to minimize systemic error [11]. The ONT sequencer generates sequencing reads of more than 2 million base pairs from a DNA or RNA template. The ONT platform allows sequencing of the entire 1532-bp-long 16S rRNA gene, which would allow more accurate identification of bacterial communities [12]. Moreover, direct sequencing of nucleic acids lessens the influence of high guanine-cytosine (GC) contents or highly repetitive regions with PCR-based sequencing [13].

In this study, the fecal microbiotas of healthy subjects (*n* = 53), patients with adenomatous polyps (*n* = 43), and immunochemical fecal occult blood test (iFOBT)-positive patients (*n* = 36) were classified using short-read (MiSeq, Illumina, CA, USA) and long-read platforms (MinION, ONT, Oxford, UK). Herein, we found significant differences in *Klebsiella* and *Fusobacterium* genera within the gut microbial communities of patients with adenomas compared to those of healthy subjects, which was not noted between the OB-positive and healthy groups. Taken together, identification of the polyp-related microbial composition using high-throughput sequencing or a derived strategy has the potential to function as an auxiliary test toward the early prediction of CRC occurrence.

## 2. Materials and Methods

### 2.1. Ethics Statement of Clinical Enrollments

The recruitment procedure of clinical participants was reviewed and approved by the Joint Institutional Review Board of Taipei Medical University (TMU; approval no. 201901013). Clinical participants were enrolled from the Division of Colorectal Surgery at Taipei Medical University and the Department of Family Medicine at Wan Fang Hospital (both in Taipei, Taiwan). Taking antibiotics, a history of chemotherapy or radiation therapy, and regular use of a feces softener within 3 months were the exclusion criteria for enrollment.

### 2.2. Bacterial DNA Extraction

Feces samples were reserved in DNA/RNA Shield Fecal Collection tubes (Zymo Research, Irvine, CA, USA). Genomic DNAs of gut microbiota were extracted using a Quick-DNA Fecal/Soil Microbe Microprep Kit (Zymo Research, Irvine, CA, USA) according to the manufacturer’s instructions. The quantity and purity of the genomic DNA samples were examined using a fluorometric assay (GeneCopoeia, Rockville, MD, USA). Extracted genomic DNA samples were stored in a 80 °C freezer until used.

### 2.3. 16S rRNA Gene Sequencing

To conduct short-read sequencing, 10 ng of the genomic DNA sample was subjected to library construction using a Nextera XT Library Prep. Kit (Illumina, CA, USA) according to the manufacturer’s protocol. Bridge amplification was conducted using the Miseq Reagent kit v3 for 600 cycles (Illumina, CA, USA) with the Illumina Miseq platform. The total number of generated reads was 50,000–100,000 on average per sample. To perform long read-sequencing, 10 ng of the genomic DNA sample was subjected to library construction using a 16S Barcoding kit (SQK-16S024; ONT, Oxford, UK) according to the manufacturer’s protocol. Barcoded library pools were sequenced on MinION flow cells (FLO-MIN106D R9.4.1, ONT, Oxford, UK). The total number of sequenced reads was 30,000–100,000 per sample after 17 h, and the length of a sequenced read was 1532 nt on average.

### 2.4. Bioinformatic Analysis

Short reads generated and sequenced using the MiSeq platform were analyzed using the Microbial Genomics Module of the CLC Genomics Workbench (v20.0.4; CLC bio, Aarhus, Denmark). Filtered and trimmed reads were aligned to a 16S rRNA reference curated on the SILVA reference (v.128). Taxonomic profiling and find best matches with K-mer Spectra (Microbial Genomics Module; CLC Genomics Workbench, Aarhus, Denmark) were applied for taxa identification. The long-read sequencing was processed via EPI2ME (https://epi2me.nanoporetech.com), a cloud-based algorithm for classification of 16S rRNA with MinION results. MinION-generated reads were uploaded and accessed through a web interface. The analytical results generated using EPI2ME showed classification of 16S rRNA mapped to the NCBI database, which contains 18,927 16S rRNA references. MinION data were synchronously accessed using the Microbial Genomics Module (CLC Genomics Workbench, Aarhus, Denmark) with SILVA v128 references to generate the taxonomic profiles.

### 2.5. Statistical Analysis

Detailed descriptions of short-read or long-read sequencing results, including the total read number, read quality, and coverage rate obtained by MiSeq and MinION sequencing, are shown as the mean ± standard error of the mean (SEM). Continuous variables were calculated using a one- or two-way analysis of variance (ANOVA) followed by Tukey’s multiple-comparison post-hoc test. Variables were significant with a *p*-value of <0.05 (* *p* < 0.05; ** *p* < 0.01; *** *p* < 0.005). Recruitment of 50 participants per group was sufficient to see a moderate effect size (0.60–0.08) with a significance of 5% and statistical power of 80% [14]. The differential abundance of identified OTUs between groups at the species level was analyzed using the linear discriminant analysis (LDA) effect size (LEfSe) method with default settings through the website interface (https://huttenhower.sph.harvard.edu/galaxy/root). The gut microbiotas were considered significantly different with a *p*-value < 0.05 and an LDA score (log10) > 3. The utility of LDA-selected taxa or iFOBT results for predicting a diagnosis of adenomatous polyps was evaluated using the receiver operating characteristic (ROC) curve and area under curve (AUC) ratio using R programming.

## 3. Results

### 3.1. Metadata of Recruited Subjects in This Study

In total, 43 patients with polyps were recruited as the case group, 36 patients with iFOBT-positive results were recruited as the OB group, and 53 healthy subjects were included as the healthy group in this study. Histological examinations revealed that the majority of the case group had adenomatous polyps, while the minority had adenomatous coupled with hyperplastic polyps. No differences in the following confounders of age, sex, or a history of smoking or drinking were noted among the case, iFOBT-positive, and healthy groups (Table 1, *p* > 0.1).

### 3.2. Overview of Gut Microbial Communities in Recruited Participants Evaluated by Short- and Long-Read Sequencing Results

In this study, a short-read sequencer (MiSeq, Illumina, CA, USA) and a long-read sequencer (MinION, ONT, Oxford, UK) were synchronously applied to classify fecal microbial communities of the same batch of DNA samples. Total numbers of qualified short and long reads generated with an average of short or long reads per sample using distinct sequencing platforms were filtered using CLC Genomics Workbench (v.20, Aarhus, Denmark) and are shown in Table 2.

**Figure 1:**
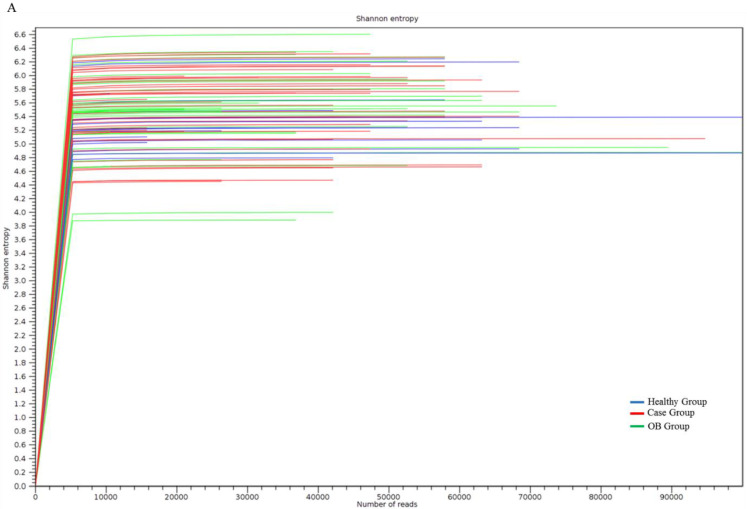

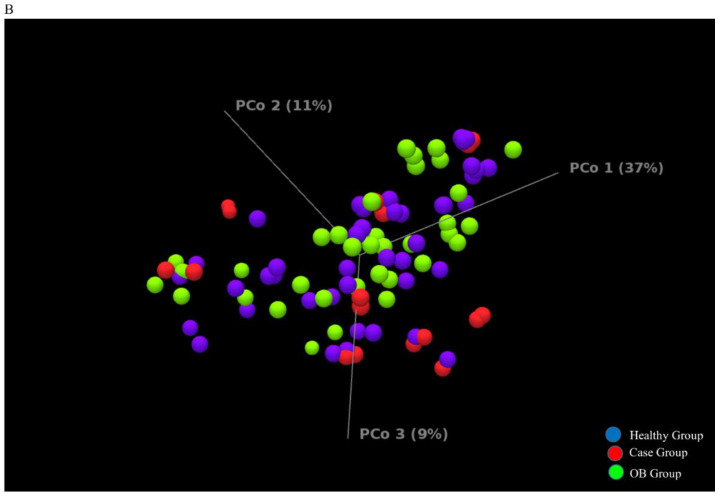
Diversity of taxonomic identification between three groups of participants. (**A**) The α-diversity in all groups was estimated using Shannon indices. (**B**) Weighted Unifrac principal component analysis (PCA) was performed to evaluate the β-diversity in all groups of participants.

According to the results of the Shannon index, no significant difference in terms of α-diversity was identified between the groups’ gut microbiotas using the MiSeq sequencer (Figure 1A). The results of a weighted UniFrac principal coordinate analysis (PCoA) showed that no differential aggregation was identified between iFOBT-positive subjects (Figure 1B, green dot) and the healthy group (Figure 1B, blue dot), whereas unique aggregates were identified in case subjects (Figure 1B, red dot). These results demonstrated a difference in the composition, but not the richness or diversity, of gut microbiotas between the case group and the other participants.

### 3.3. Comparison of Gut Microbiota in iFOBT-Positive Patients and the Healthy Group Using Distinct Sequencing Platforms

**Figure 2:**
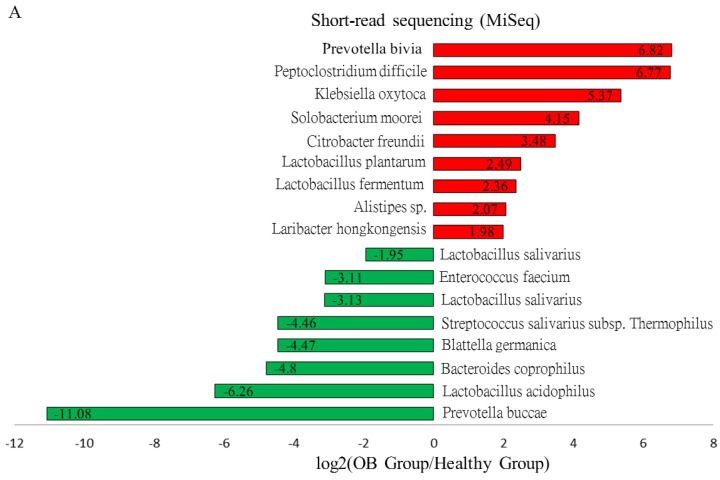

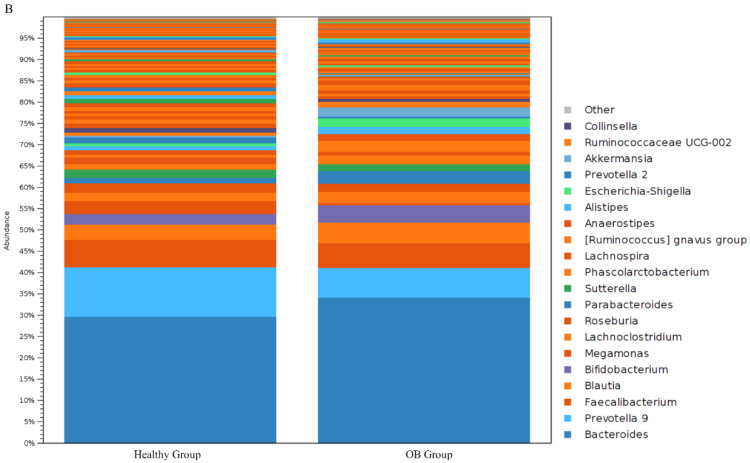
Distinct composition of microbial community of the healthy group and the OB group with short-read sequencing results. (**A**) Increases (red bar) or decreases (green bar) in the differential abundance of identified taxa in OB group as compared to the healthy group. (**B**) The relative abundances of top 20 classified taxa in two groups.

The iFOBT test has long been applied as an early prediction approach toward CRC development [7]. In this study, gut microbial analyses using a high-throughput sequencing platform functioned as an auxiliary approach for evaluating the gut environment between iFOBT-positive and healthy subjects. The results of a differential abundance analysis showed that decreases in relative levels of *Bacteroides*, *Lactobacillus*, and *Prevotella* genera were identified in the gut microbiota of iFOBT-positive subjects compared to the healthy group (Figure 2A, green bar, *p* < 0.001) using the MiSeq platform. No substantial difference in the relative abundance of potential pathogens was found from results of the abundance analysis. The compositions of the top 20 classified OTUs at the genus level in the two groups with short-read sequencing results are presented in Figure 2B.

**Figure 3:**
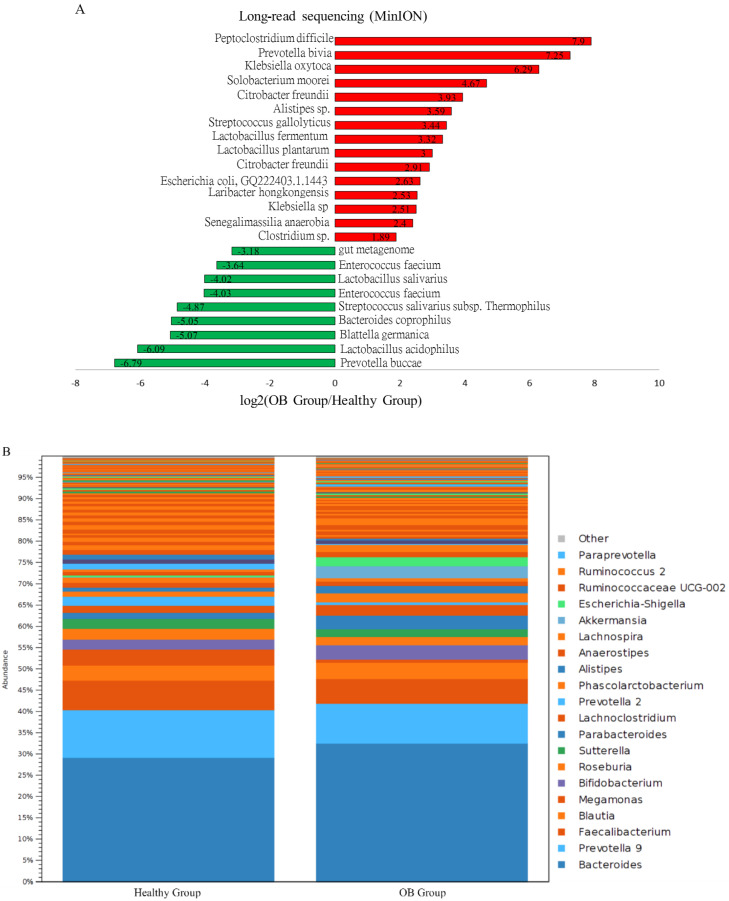
Distinct composition of microbial community of the healthy group and the OB group with long-read sequencing results. (**A**) Increases (red bar) or decreases (green bar) in the differential abundance of identified taxa in OB group as compared to the healthy group. (**B**) The relative levels of top 20 classified taxa in two groups with MinION results.

It was demonstrated that long-read sequencing had a higher efficiency than short-read sequencing in taxonomic classification of gut microbiota at the species level [12]. In this study, over 250 taxa at the genus level, or 700 taxa at the species level, were classified into individual groups using MinION sequencing coupled with the EPI2ME algorithm or the Microbial Module of CLC Genomics Workbench. Taxa dominantly abundant in the healthy and OB-positive groups included *Blautia*, *Faecalibacterium*, *Bacteroides*, and *Prevotella* genera (Figure 3B), which were consistently identified with MiSeq results (Figure 2B). The results of the differential abundance assays indicated decreases in the relative levels of *Enterococcus*, *Lactobacillus*, *Prevotella*, and *Bacteroides* with concomitant increases in the relative abundances of *Klebsiella*, *Streptococcus*, *Clostridium*, and *Citrobacter* in the iFOBT-positive group compared to the healthy group (Figure 3A). The alterations in the gut microbial community of the OB community compared to the healthy group were mostly identified using a distinct sequencing platform.

### 3.4. Characterization of Adenomatous-Polyp-Related OTUs in the Case Group Using Distinct Sequencing Platforms

**Figure 4:**
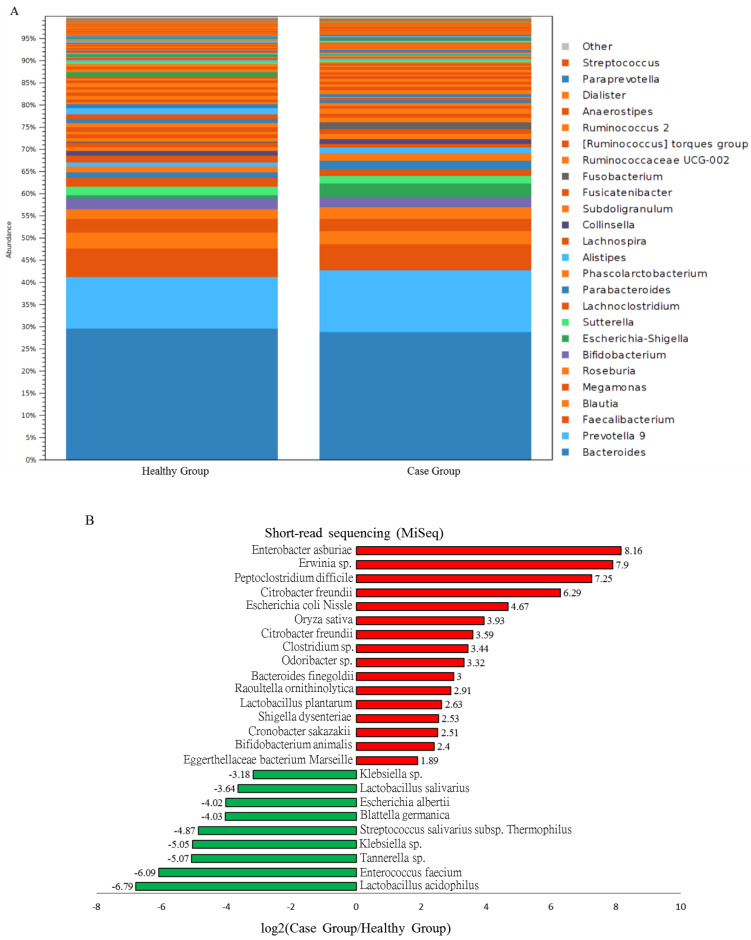

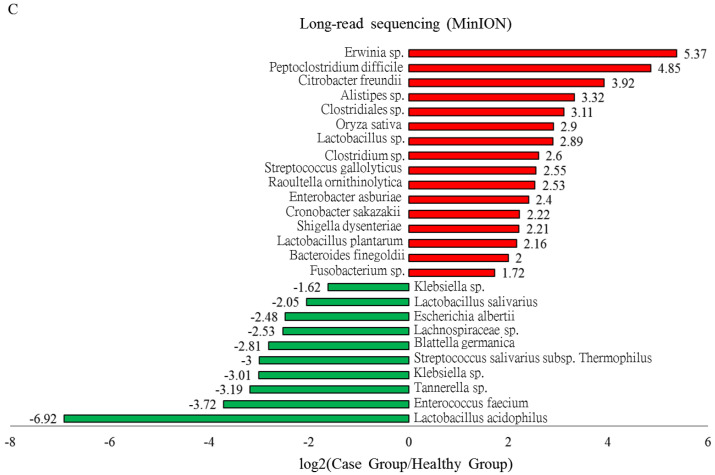
Distinct composition of microbial community of the healthy group and the case group with short- and long-read sequencing results. (**A**) The relative levels of top 25 classified taxa in these two groups with short-read sequencing data. (**B**) Increases (red bar) or decreases (green bar) in the differential abundance of identified taxa in Case group as compared to the healthy group with MiSeq data. (**C**) Increases (red bar) or decreases (green bar) in the differential abundance of identified taxa in Case group as compared to the healthy group with MinION results.

A growing body of studies has demonstrated differential profiles of the gut microbiota in the healthy population and the clinical participants with adenomatous polyps using a distinct analytic workflow. In this study, the results of MiSeq sequencing coupled with the Microbial Module analysis (CLC Genomics Workbench) showed that the high abundances of the *Bacteroides*, *Prevotella*, *Faecalibacterium*, and *Bifidobacterium* genera were predominantly assigned to the bacterial communities of case and healthy groups in this study (Figure 4A). Statistically significant increases in the relative abundances of pathogenic bacteria, including *Escherichia*-*Shigella*, *Klebsiella*, and *Enterobacter* genera, were identified through the abundance analyses of the gut microbiota of subjects with adenomatous polyps (Figure 4B, red bars). The classified taxa that differed in relative abundances between participants with adenomatous polyps and healthy subjects, including the *Shigella*, *Streptococcus*, and *Fusobacterium* genera, were noted with the MinION sequencing results (Figure 4C, red bars). Taken together, the taxa that differed in relative abundances between participants with adenomatous polyps and healthy subjects at the generic level were consistently identified using short- or long-read sequencing analyses in this study.

### 3.5. Identified OTUs at the Species Level that Differed between the Case Group and Healthy Participants Classified Using MinION Sequencing

**Figure 5:**
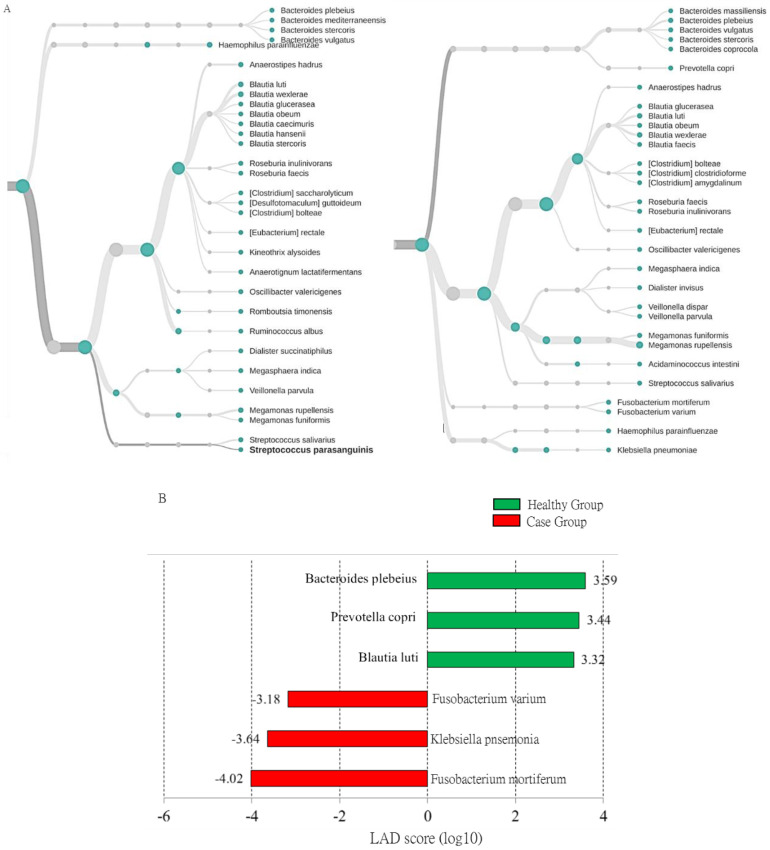
Characterization of identified taxa in adenomatous polyp participants and healthy subjects using LEfSe analysis. (**A**) The relative abundances of the top 30 classified OTUs to species level in healthy group (left) and adenomatous group (right) with MinION data. (**B**) Histogram of the LDA scores computed for OTUs with differential abundance in the healthy subjects and the participants with adenomatous polyp (case group).

We further assessed the utility of the MinION results for classifying the identified taxa at the species level in the case group and healthy participants. As shown in Figure 5A, the taxonomy tree presented the top 30 classified OTUs to the species level in the healthy (Figure 5A, left) and case groups (Figure 5A, right) with the MinION results. The differential abundance of identified taxa between the healthy and case groups was estimated using the linear discriminant analysis (LDA) effect size (LEfSe) assay. The LDA scores indicated relatively more abundant levels of *Fusobacterium mortiferum*, *Fusobacterium varium*, and *Klebsiella pneumonia* in participants with adenomatous polyp (case group) than in the healthy group (Figure 5B; LDA score (log10) > 3). In contrast, *B. luti*, *B. plebeius*, and *P. copri* were enriched in the gut microbiota of the healthy group (Figure 5B, green bars). The presence of these taxa relative to all microbial communities was relatively abundant (more than 1%).

**Figure 6:**
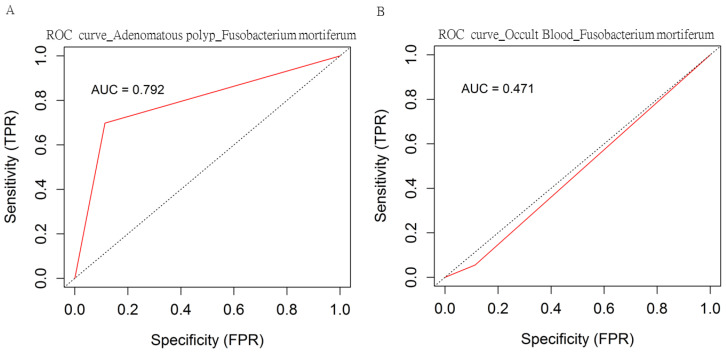
Area under the ROC curve (AUC; red line: ROC curve) for the prediction of (**A**) adenomatous polyp or (**B**) occult blood based on the relative abundance of *F. mortiferum* in fecal samples with the MinION sequencing results.

The potential utility of the gut microbiota as a biomarker for the occurrence of adenomatous polyps was next evaluated. The high relevance between adenomatous polyps and identified taxa was evaluated using the receiver operating characteristic (ROC) curve. As shown in Table 3, the relatively high abundances of *K. pneumonia* and *F. mortiferum* might be more predictive of the presence of adenomatous polyps than other OTUs classified in this study. The ROC curve generated with the relative abundance of *F. mortiferum* toward the diagnosis of adenomatous polyps resulted in an area under the curve (AUC) of 0.792 (Figure 6A), whereas the ROC curve generated with the same predictive taxa toward iFOBT-positive resulted in an AUC of 0.471 (Figure 6B). Taken together, an increase in the relative abundance of *F. mortiferum* could be considered an emerging and auxiliary biomarker for the presence of adenomatous polyps.

## 4. Discussion

In the present study, differences in the gut microbiota down to the species level of patients with adenomatous polyps, but not OB-positive participants, were classified using a long-read sequencing platform. In total, differential abundances of six OTUs were identified between patients with adenomatous polyps and a healthy population. These taxa were predictive of and associated with the presence of adenomatous polyps.

A correlation between the enrichment of *Fusobacterium* spp., especially *F. nucleatum*, and the advancement of CRC has been long reported with the application of cancerous tissues or fecal samples [15]. *F. nucleatum* was also reported to account for 60–80% of all *Fusobacterium* spp. in both healthy participants and patients with adenomatous polyps [16]. These results suggest the diverse potential impacts of *F. nucleatum* on healthy cohorts and CRC patients. In this study, the relatively high abundance of *F. nucleatum* was not identified in all polyp-containing participants with short-read sequencing and long-read results. In contrast, differential abundances of *F. varium* and *F. mortiferum* were found in most patients with adenomatous polyps, but not in the healthy group or the OB-positive group. The dominant presence of *F. varium* was demonstrated to activate host inflammatory signaling by invading or adhering to the epithelial layer of the gut [17]. The genomic sequence of *F. varium* was previously revealed in the colon mucosal membrane of patients with ulcerative colitis, which manipulated expressions of particular proteins [18]. These results suggest the potential influence of *F. varium* on chronic inflammation, the occurrence of polyps, and CRC development. An increase in the relative abundance of *F. mortiferum* was also identified in the adenomatous-polyp-containing patients with the mutant *adenomatous polyposis coli* (*APC*) gene, though interactions and correlations of *F. mortiferum* with unique genes are worthy of further investigation. [19]. Taken together, *Fusobacterium* spp. exhibited diverse impacts on distinct stages of CRC development.

In addition to CRC-related or adenomatous-polyp-associated taxa, alterations in the normal flora or probiotics may function as another biomarker for early prediction or screening. For instance, in vivo and in vitro studies demonstrated the beneficial effects of the Prevotellaceae family toward the gut environment [20]. A 40-fold higher abundance of *Prevotella copri* was identified in the gut microbial community isolated from healthy subjects compared to CRC patients [21]. *Prevotella* spp. was reported to generate butyrate, which is potentially capable of repressing inflammatory signals involved in carcinogenesis [22]. Interestingly, increases in the relative abundances of *P. copri* and *B. plebeius* were noted in CRC patients treated with 5′-fluorouracil chemotherapy [23,24]. However, abundances of *Prevotella*, *Bacteroides*, and *Blautia* spp. were significantly altered by dietary intake [25]. A comprehensive cohort investigation is required to further assess the influence of potential probiotics and the normal flora on the occurrence of adenomatous polyps.

## 5. Conclusions

Sequencing of the v3–v4 region within *16S rRNA* using the Illumina platform (MiSeq) has been widely applied in studies in the microbial field [26]. In contrast, the long-read ONT platform (MinION) allows sequencing of the entire *16S rRNA* gene via a non-PCR-based approach [27], which diminishes the generation of PCR-synthesis-mediated bias. MinION results were simply analyzed using an ONT-developed EPI2ME workflow with the NCBI 16S reference database, which enabled the taxonomic classification down to the species level [27]. ONT provides an interesting and alternative solution to mitigate the aforementioned issue regarding classification of microbial communities with the *16S rRNA* gene. In this study, identification of adenomatous polyp-associated microbiomes could potentially function as an auxiliary biomarker for predicting CRC development.

## Figures and Tables

**Table 1:** Demographics of the healthy, iFOBT-positive, and adenoma groups.

	Healthy Group (*n* = 53)	FOBT-Positive (*n* = 36)	Adenomatous Polyp (*n* = 43)	*p*
Age (Median(IQR))	64 (33–69)	53 (47–55)	56 (43–59)	0.63
Sex (*n*,%) Female Male	31 (58.5) 22 (41.5)	20 (55.56) 16 (44.44)	20 (46.51) 23 (35.49)	0.58
History of cancer (*n*,%)	6 (11.32)	3 (8.33)	5 (11.63)	0.98
Family history of cancer (*n*,%)	11 (20.75)	6 (16.67)	10 (23.26)	0.56
History of smoking (*n*,%)	15 (28.3)	8 (22.22)	13 (30.23)	0.52
History of drinking (*n*,%)	4 (7.54)	5 (13.89)	8 (18.6)	0.12
History of regular exercise (*n*,%)	24 (45.28)	17 (47.22)	20 (46.51)	0.73

**Table 2:** Identified results of short-read amplicons or long-read sequencing with corresponding database for taxonomic assignment at genus or species levels.

Sequencing Platform		Miseq						MinION			
Number of Raw reads (*n* = 132)	Number of classified reads (*n* = 132)	Genus		Species		Number of Raw reads (*n* = 132)	Number of classified reads (*n* = 132)	Genus		Species	
		CC	UC	CC	UC			CC	UC	CC	UC
9,404,348	8,511,812	95.57%	4.43%	71.29%	28.71%	7,094,472	6,810,408	97.32%	2.68%	76.83%	23.17%

Abbreviations: CC: correctly classified; UC: unclassified.

**Table 3:** Statistical results of identified taxa with MinION results to the species level in the adenomatous polyp and healthy groups.

	Relative Abundance (Case Group/Healthy Group)	*p*-Value
Sample No.	Case Group (43)/Healthy Group (53)	
*Fusobacterium Mortiferum* (Fold change; mean (SD))	10.182 (3.41)	<0.001
*Klebsiella Pneumonia* (Fold change; mean (SD))	15.286 (4.91)	<0.001

## Abbreviations

NGS	Next generation sequencing
ONT	Oxford nanopore technology
iFOBT	immunochemical fecal occult blood test
